# Deep Convolutional Approaches for the Analysis of COVID-19 Using Chest X-Ray Images From Portable Devices

**DOI:** 10.1109/ACCESS.2020.3033762

**Published:** 2020-10-26

**Authors:** Joaquim De Moura, Lucía Ramos García, Plácido Francisco Lizancos Vidal, Milena Cruz, Laura Abelairas López, Eva Castro Lopez, Jorge Novo, Marcos Ortega

**Affiliations:** 1 Centro de Investigación CITICUniversidade da Coruña 16737 15071 A Coruña Spain; 2 Grupo VARPAInstituto de Investigación Biomédica de A Coruña (INIBIC), Universidade da Coruña 16737 15006 A Coruña Spain; 3 Servicio de RadiodiagnósticoComplexo Hospitalario Universitario de A Coruña (CHUAC) 15006 A Coruña Spain

**Keywords:** Chest X-ray imaging, COVID-19, deep learning, X-ray portable device

## Abstract

The recent human coronavirus disease (COVID-19) is a respiratory infection caused by severe acute respiratory syndrome coronavirus 2 (SARS-CoV-2). Given the effects of COVID-19 in pulmonary tissues, chest radiography imaging plays an important role in the screening, early detection, and monitoring of the suspected individuals. Hence, as the pandemic of COVID-19 progresses, there will be a greater reliance on the use of portable equipment for the acquisition of chest X-ray images due to its accessibility, widespread availability, and benefits regarding to infection control issues, minimizing the risk of cross-contamination. This work presents novel fully automatic approaches specifically tailored for the classification of chest X-ray images acquired by portable equipment into 3 different clinical categories: normal, pathological, and COVID-19. For this purpose, 3 complementary deep learning approaches based on a densely convolutional network architecture are herein presented. The joint response of all the approaches allows to enhance the differentiation between patients infected with COVID-19, patients with other diseases that manifest characteristics similar to COVID-19 and normal cases. The proposed approaches were validated over a dataset specifically retrieved for this research. Despite the poor quality of the chest X-ray images that is inherent to the nature of the portable equipment, the proposed approaches provided global accuracy values of 79.62%, 90.27% and 79.86%, respectively, allowing a reliable analysis of portable radiographs to facilitate the clinical decision-making process.

## I. Introduction

Coronavirus disease 2019 (COVID-19) is a respiratory infection caused by the novel coronavirus severe acute respiratory syndrome coronavirus 2 (SARS-CoV-2) [1]. It initially emerged in late December 2019 as a cluster of cases of pneumonia with unknown cause, rapidly spreading worldwide [2]. Given the alarming levels of spread across the world and its severity, the World Health Organization (WHO) declared the outbreak of COVID-19 as a global pandemic on 11 March 2020, with more than 118,000 confirmed cases, including 4,291 deaths reported in 114 countries [3]. By August the 29th 2020, it had affected more than 213 countries/regions, with 24,537,560 confirmed cases, including 832,879 deaths, reported to the World Health Organization (WHO) [4].

Among the observable symptoms, the infection can manifest fever, coughing, shortness of breath, pneumonia, and acute respiratory distress syndrome, with severe cases associated with intensive care unit admission and high mortality [5]. The severity of the disease is related to advanced age and co-morbidities such as chronic heart and lung disease [6]. Additionally, the virus is highly contagious, spreading efficiently by human to human transmission via droplets or close contact [7], with an $\text{R}0\geq3$, that is, on average, each infected person is capable of transmitting the virus to 3 other people [6]. Therefore, the COVID-19 is considered as a public health emergency, and according to the key areas described by the WHO, the early diagnosis is a priority in order to break the chain of transmission and, therefore, control the spread of the virus [4].

During the last decades, chest radiography imaging has been a diagnostic tool commonly used in the clinical practice to examine abnormalities in the cardiothoracic region, in order to detect and monitor the evolution of several pulmonary diseases, such as emphysema, chronic bronchitis, pulmonary fibrosis, lung cancer or pneumonia, among others [8]–[11]. Regarding the COVID-19 disease, several manifestations and patterns of lung abnormality were identified in chest X-ray images such as bilateral abnormalities, interstitial abnormalities, lung consolidation, and ground-glass opacities [12]. Therefore, the analysis of chest X-ray images of the suspected individuals presents a remarkable potential for screening processes and early diagnosis of COVID-19 disease [13], even more when it is known that Polymerase Chain Reaction (PCR) studies, despite their effectiveness, have a percentage of false negatives, being therefore the radiological analysis another complementary tool to support and confirm the diagnosis of these patients [14]. In this context, the specialist’s experience is essential for the identification and characterization of biomarkers associated with COVID-19, as well as the differentiation from other clinical findings related to other respiratory diseases with similar characteristics, as pneumonia. However, the subjective appreciation of the clinical biomarkers and the variety of X-ray images entail an inter and intra expert variability that affects the repeatability of the manual evaluation of chest X-ray images. Besides the subjectivity, the manual characterization is a tedious and time-consuming task. Therefore, a fully automatic system for the analysis of chest X-ray images identifying normal, pathological or specific COVID-19 cases would reduce the workload of the clinical staff allowing a reliable and repeatable evaluation to support the clinical decision-making process.

Over recent years, deep learning techniques have demonstrated their potential, effectiveness, and versatility, positioning themselves as benchmarks in solving a large number of problems in multiple fields, highlighting computer vision. The advances in terms of computational capabilities as well as the availability of large labeled datasets have promoted the use of deep learning techniques in the field of medical imaging [15]. In particular, the potential of these techniques has been exploited for the detection and assessment of cardiothoracic and pulmonary abnormalities in chest X-ray imaging, one of the most widely used imaging tests in medical practice [16], [17]. Therefore, Anthimopoulos *et al.* [18] present a deep CNN (Convolutional Neural Network) to classify lung image patches into 7 categories considering 6 different patterns of interstitial lung diseases as well as healthy tissue. In the work of Campo *et al.* [19], the authors present a method based on a CNN for the quantification of the emphysema percentage on simulated chest X-ray images. Jaiswal *et al.* [20] propose a deep neural network that incorporates global and local features for the identification of pneumonia in chest X-ray images. Pasa *et al.* [21] explore deep network architectures tailored to tuberculosis diagnosis. Other works in this field incorporate transfer learning techniques in order to perform an effective learning stage over a limited dataset using transferring features extracted from other larger datasets in the same domain. Thus, Ausawalaithong *et al.* [11] presents a method based on a DenseNet-121 along with multi-transfer learning in order to classify lung cancer in chest X-ray images. The method consists of a first stage aimed at transferring the model from general image domain to chest X-ray domain, allowing the classification between chest X-ray images with nodules or without nodules. After that, the second stage of transfer learning is used for the specialization of lung cancer, allowing the classification between chest X-ray images with malignant nodules or without malignant nodules. The entire process provides a model more specific for the lung cancer assessment. In the work of Chouhan *et al.* [22], the authors present a novel ensemble approach based on the transfer learning of five different neural network architectures.

In the context of the global pandemic originated by the COVID-19 disease, given the relevance of the topic, during the last months, several authors proposed computational approaches for the COVID-19 classification on chest X-ray images using deep learning techniques. As reference, Apostolopoulos *et al.* [23] explore the possibility of extracting representative COVID-19 biomarkers from chest X-ray images by means of deep learning strategies. In the work of Wang *et al.* [24], the authors present COVID-Net, a deep convolutional neural network tailored for detecting COVID-19 cases from chest X-ray images. Hammoudi *et al.* [25] proposed a deep learning method applied to chest X-ray images to identify health and pneumonia cases, differentiating between bacterial or viral origin, assuming that, during the epidemic season, if the origin of pneumonia is viral, there is a high probability of being a positive COVID-19 case. Hemdan *et al.* [26] proposed COVIDX-Net, an approach comprising seven convolutional neural networks for the diagnosis of COVID-19 in chest X-ray images. De Moura *et al.* [27] presented several fully automatic deep convolutional approaches for the classification and study of COVID-19, common pneumonia and healthy chest X-ray images. In the work of Zhang *et al.* [28], the authors proposed a deep anomaly detection model for COVID-19 screening on chest X-ray images. Ozturk *et al.* [29] proposed a deep learning model that provides heatmaps highlighting important areas in the chest X-ray images to help the specialists to locate the affected regions for the early detection of the COVID-19 disease. Shelke *et al.* [30] present a method for the classification of chest X-ray images into the classes normal, pneumonia, tuberculosis, and COVID-19. In the case of COVID-19, additional categorization into mild, medium, and severe is also provided. In the work of Yoo *et al.* [31], the authors propose a deep learning-based decision-tree classifier for the detection of COVID-19 in chest X-ray images. It is composed of a first decision tree that classifies the images as normal or abnormal, then, a second tree identifies the abnormal images that present signs of tuberculosis, whereas the third tree identifies the clinical findings related to COVID-19. Li *et al.* [32] explore the use of a convolutional siamese neural network-based algorithm for the estimation of pulmonary disease severity on chest X-ray images from patients with COVID-19 in order to predict subsequent intubation or death. This method can also be used for longitudinal disease evaluation and risk stratification. In the work of Khan *et al.* [33], the authors present CoroNet, a deep learning method for the automatic COVID-19 detection in chest X-ray images considering the binary classification COVID-19 vs no-findings and the multi-class classification into the categories no-findings, pneumonia, and COVID-19. Data augmentation and transfer learning techniques are also used in order to overcome the limited size of some datasets and take the most use of the available data. Therefore, in the work of Waheed *et al.* [34], the authors present CovidGan, a method based on a Classifier Generative Adversarial Network that allows generating synthetic chest X-ray images in order to enhance the performance of a Convolutional Neural Network for COVID-19 detection. Narin *et al.* [35] evaluates three different approaches using convolutional neural networks based on transfer learning for the detection of pneumonia associated with COVID-19 in the chest X-ray images. In the work of Dansana *et al.* [36], the authors proposed an automatic methodology for the early diagnosis of COVID-19 patients using X-ray images of the chest and CT scan images collected from different sources. Minaee *et al.* [37] explores 4 state-of-the-art convolutional networks for the COVID-19 detection using transfer learning over a combined dataset generated from different publicly available datasets. In the work of Basu *et al.* [38], the authors present a method based on transfer learning called Domain Extension Transfer Learning that tunes a model pre-trained on a related large chest X-ray dataset for the classification into the classes normal, other diseases, pneumonia, and COVID-19.

Although the methods that were proposed in the literature provided good results, most of these approaches use a very small dataset, COVID-ChestXRay [39], which consists of 123 COVID-19 cases. In particular, these images were obtained from highly heterogeneous equipment from all around the world. As a consequence of having a very little and heterogeneous data set, the test set sizes are extremely limited and do not provide any statistical certainty about the learning. In addition, to build negative COVID-19 cases, data samples are typically taken from other datasets (mostly, from the RSNA Pneumonia CXR challenge dataset on Kaggle [40]). These images were obtained from the same X-ray equipment. In this case, a deep learning model can group the X-ray images according to the scanning equipment used for the examination instead of the clinical findings associated with the different conditions.

Furthermore, these datasets are mostly composed of images captured by fixed X-ray equipment. The quality of the chest X-ray images in terms of spatial resolution, contrast, presence of artifacts, or noise is conditioned by the capture equipment and the acquisition technique [41], [42]. The nature of fixed equipment allows the optimization of parameters such as the exposure time, the radiation field size, or the orientation and distance of the X-ray tube, maximizing the details of the perceived information content [41], [43]. However, in the context of the global pandemic caused by COVID-19, a properly decontamination of the equipment used for the acquisition of the chest X-ray images as well as an effective control during the patient transport to the X-ray room are critical factors in controlling the transmission of the virus [44]. The use of portable equipment for the acquisition of chest X-ray images allows to minimize the patient transportation facilitating a more effective separation of suspected patients with other low-risk cases [45]. In this sense, the American College of Radiology (ACR) recommends the use of portable chest X-ray acquisition equipment in order to minimize the risk of cross-infection given the ease of decontamination of the surface of portable devices and the capability of acquiring the chest X-ray images in isolation areas, avoiding the X-ray room or common areas such as waiting rooms or hallways [46]. Although the use of portable equipment entails a significant penalization in the quality of the captured images [47], it has been the first and main radiological analysis procedure in order to detect possible findings that support the diagnosis of suspected patients, as well as determine its severity, minimizing the risk of virus transmission. Additionally, portable devices can also be very effective for the triaging of patients with early symptoms but instructed to stay at home if there are no severe signs, thus avoiding the risk of the virus spreading and the collapse of hospital centers. Furthermore, as the COVID-19 pandemic progresses, there will be a greater reliance on portable devices given their accessibility, widespread availability as well as the reduced decontamination issues required for an adequate use [44].

Given the great relevance and utility of the portable equipment to face the public health emergency caused by the COVID-19 pandemic, in this work, we propose a comprehensive methodology and derived accurate approaches for the analysis of COVID-19 in the chest X-ray images acquired by portable devices. To the best of our knowledge, it is the first fully automatic methodology for the identification of COVID-19 in the chest X-ray images acquired exclusively through portable equipment. It is worth noting that the use of portable units implies a greater challenge for the automatic diagnostic of the COVID-19, given that the quality of the chest X-ray images in terms of spatial resolution, contrast, or amount of perceived information is limited by the nature of the portable equipment as well as the acquisition procedure involved in this type of devices.

The proposed paradigm arises from the difficulty of differentiating specific cases of COVID-19 from other respiratory affections and lung diseases that present similar clinical conditions. In this context, a dataset has been specifically designed for this analysis, including cases with COVID-19, cases with lung conditions different from COVID-19, and cases without lung pathologies. Therefore, the defined dataset is divided into 3 clinical categories: *Normal*, *Pathological* and *COVID-19*. It is worth mentioning that the considered pathological cases are those from pulmonary diseases with similar findings as COVID-19, whereas the normal subjects may not be specifically healthy. In that case, they belong to healthy patients as well as other pathological cases with characteristics that differ from COVID-19. In that line, the proposed work is applied to the most complex scenario of differentiation to analyze the COVID-19 disease.

In order to emphasize the differentiation between these three categories and explore the full potential of the available dataset, the proposed methodology integrates 3 complementary approaches based on deep learning techniques. The first of these approaches is aimed at classifying between normal patients and those pathological cases that present any indication that could be related to COVID-19, either specifically related to the presence of COVID-19 or other respiratory conditions with similar symptoms. The second approach is specifically trained for the differentiation of the patients with COVID-19 with respect to patients not infected with COVID-19, whether normal cases or with respiratory conditions other than COVID-19, but with similar symptoms. Additionally, the third approach has been designed to determine the degree of separability between the 3 categories of chest X-ray images considered in this work.

This way, the response of the first approach separates healthy cases from pathological cases (including patients with COVID-19), the response of the second approach separates patients with COVID-19 from other individuals (including healthy patients), whereas the response of the third approach allows the separation between the 3 defined categories, simultaneously. Thus, the combined use of the responses from all the approaches allows reinforcing the differentiation between COVID-19, pathological patients with other similar respiratory conditions as well as other normal cases. This way, it has the potential to increase the reliability of the proposed system in the prognostic predictions to triage and manage patient care. In that sense, in the clinical collaboration contained in this contribution, the output of all approaches are simultaneously provided to the clinical specialists.

The dataset specifically designed for this study has been provided by the Radiology Service of the Complexo Hospitalario Universitario A Coruña (CHUAC) that supplied the assistance work of the radiology and computer services in order to search for adapted solutions for the improvement in the patient care. The evaluation of the prognostic performance over this dataset showed that the proposed methodology and designed approaches provide accurate results even though the images acquired by portable devices present limited quality conditions, allowing a reliable analysis to support the clinical decision-making process in the context of this dramatic global pandemic.

Summarizing, the main research highlights of the article include:

• New computational approach for the classification of chest X-ray images into 3 categories: normal, pathological, and COVID-19.
• First method specifically designed for the analysis of COVID-19 in chest X-ray images acquired exclusively by means of portable equipment.
• Methodology trained and validated over a dataset specifically designed for this research.
• Accurate results even though portable devices acquire images with poor quality conditions.

The manuscript is structured as follows: Section II presents the dataset and describes the different deep learning approaches that were proposed in this research. Next, Section III exposes all the conducted experiments whereas Section IV discusses the obtained results. Then, Section V describes the main limitations of this work. Finally, Section VI presents the conclusions that are extracted from this research as well as possible future work in the analysis of this relevant disease.

## II. Materials and Methods

### A. Used Image Dataset

The used dataset was provided by the Radiology Service of the Complexo Hospitalario Universitario A Coruña (CHUAC). The protocols for this study have been reviewed by the hospital board and are contained in an agreement with the hospital management. The images have been acquired by means of portable equipment, specifically, Agfa dr100E GE and Optima Rx200 portable devices. These portable devices were located in areas specifically intended for COVID-19 in order to examine patients referred after undergoing triage in the emergency systems and monitor these patients in the different hospital plants. The use of portable equipment allows an effective separation between infected patients and patients without suspected COVID-19, both in the radiology room itself and in waiting rooms or hallways. For the acquisition procedure, the patient lies in a supine position and a single anterior-posterior projection is recorded. For this purpose, the X-ray tube is connected to a flexible arm that is extended over the patient to be exposed to a small dose of ionizing radiation while an X-ray film holder or an image recording plate is placed under the patient to capture images of the interior of the chest.

The dataset consists of 1,616 chest X-ray images divided into 728 normal cases corresponding to patients who have neither pleural nor pulmonary pathologies (but other pathologies have not been assessed, such as cardiology or hepatic diseases, for example) at the time of image acquisition, 648 pathological cases corresponding to patients without COVID-19 but diagnosed with other pulmonary diseases that can present characteristics similar to COVID-19 i, as well as 240 specific COVID-19 cases. Therefore, this dataset provides a very challenging scenario, with a pathological category with diseases that are similar in terms of characteristics to COVID-19 and normal subjects that could present other non-related abnormalities. In any case, the dataset presents cases that are very frequent in real clinical practice scenarios.

**FIGURE 1.**
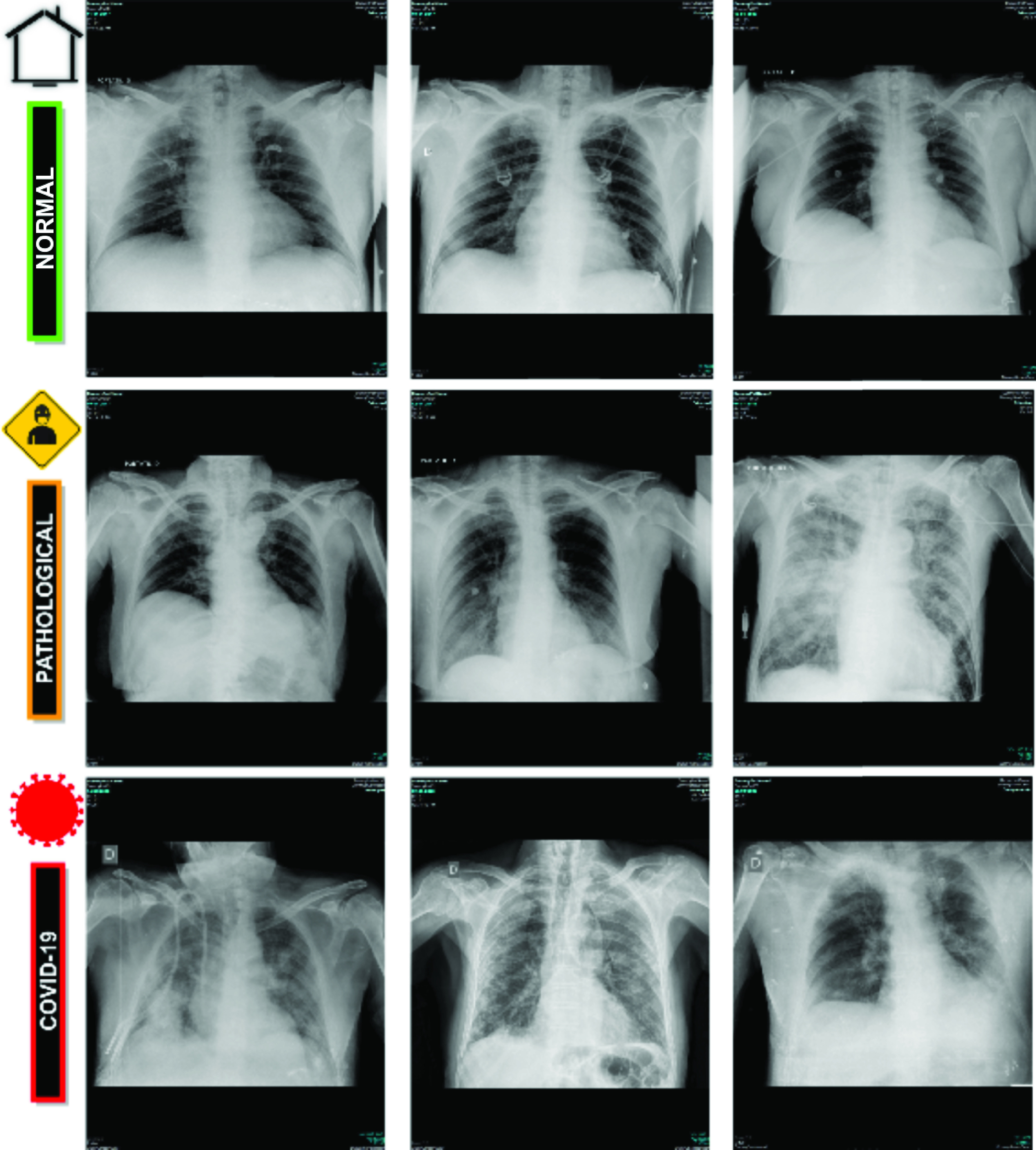
Representative examples of chest X-ray radiographs. $1^{st}$ row, chest X-ray radiographs from normal patients. $2^{nd}$ row, chest X-ray radiographs from pathological patients without COVID-19 but diagnosed with other pleural or pulmonary diseases. $3^{rd}$ row, chest X-ray radiographs from patients with COVID-19.

Fig. 1 shows representative examples of chest X-ray images related to the presented 3 clinical categories: normal, pathological, and COVID-19.

### B. Computational Approaches for the Classification of Chest X-Ray Images

The impact of COVID-19 on lung tissues can present similarities with other patterns associated with some pulmonary pathologies as common types of pneumonia, especially, during the early stages of both respiratory conditions. In order to enhance the differentiation of COVID-19 cases both from cases that present clinical findings similar to COVID-19 and from patients without respiratory conditions, 3 different clinical categories were designed and considered. Thus, the category *Normal* correspond to patients without pleural or pulmonary pathologies, the category *Pathological* is related to patients diagnosed with pleural or pulmonary pathological conditions other than COVID-19, and the category *COVID-19* is specific for COVID-19 infected patients, as stated in Section II-A.

**FIGURE 2.**
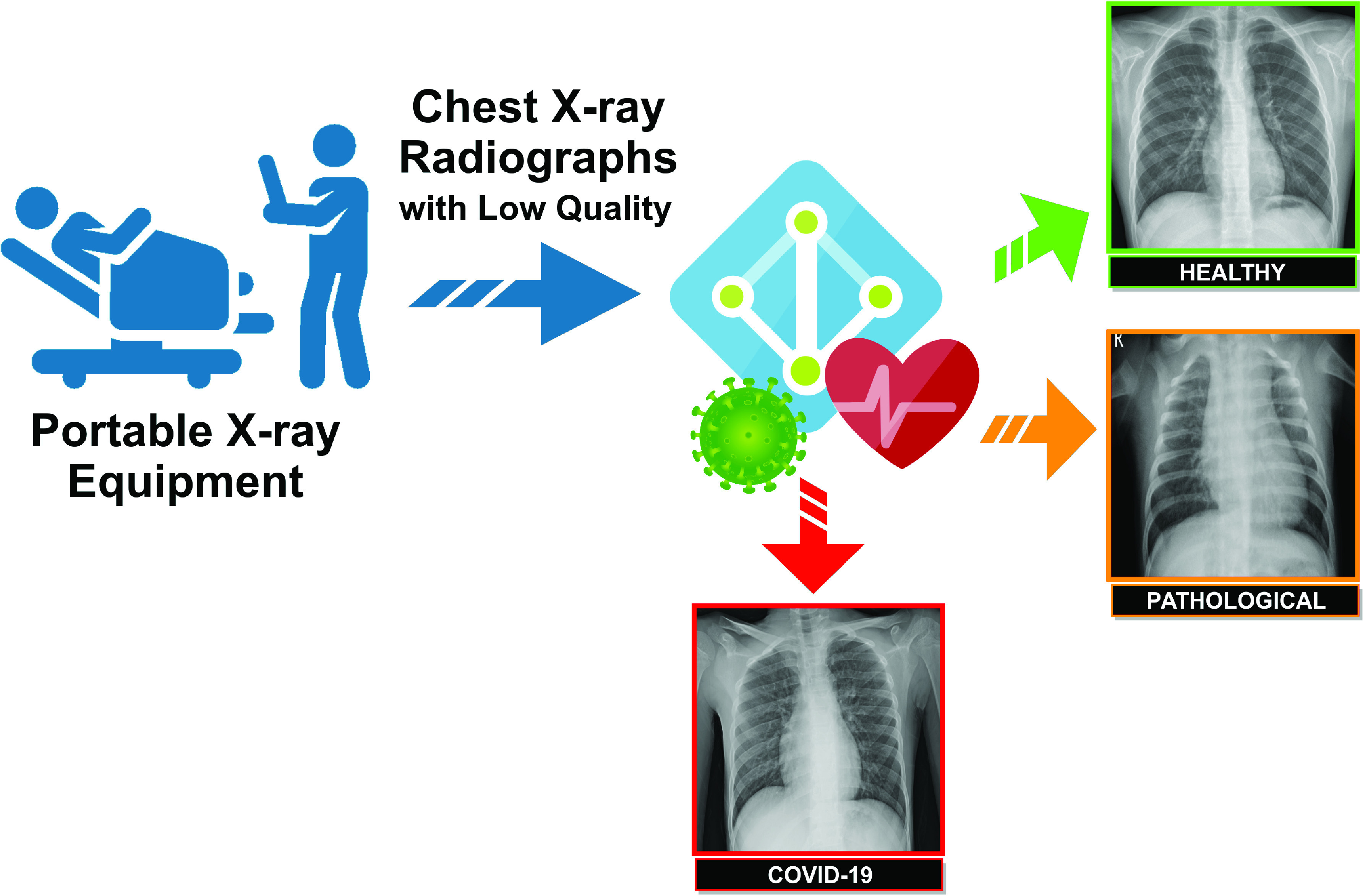
A general overview of the proposed paradigm. It receives as input chest X-ray images acquired through portable devices and provides at the output the categorization of the images into the classes *Normal*, *Pathological*, or *COVID-19*.

In order to enhance the differentiation between the defined clinical categories and exploit the full potential of the designed dataset, the proposed computational methodology integrates 3 different approaches based on deep learning techniques for the classification of chest X-ray images. A general overview of the proposed paradigm is depicted in Fig. 2. It receives a chest X-ray image captured by the portable device as input and provides its classification into the classes *Normal*, *Pathological*, or *COVID-19*. All proposed approaches, herein presented, are detailed below:

#### 1) Normal Vs Pathological/COVID-19 Approach

Given the similarities between COVID-19 and other respiratory conditions, the first trained approach is aimed at differentiating between patients with any abnormality in the pulmonary tissues and patients with clinical findings in the lungs, either due to COVID-19 or other respiratory conditions. To this end, chest X-ray images associated with pathological or COVID-19 cases are grouped under the same class for the training procedure. Therefore, this approach predicts 2 different categories: *Normal* and *Pathological/COVID-19*.

#### 2) Normal/Pathological VS COVID-19 Approach

The second trained approach is specifically intended to identify COVID-19 cases, differentiating them not only from normal cases but also from those pathological patients affected by other respiratory conditions that manifest similar characteristics, such as pneumonia. For this purpose, chest X-ray images from normal patients and those from pathological patients with respiratory conditions other than COVID-19 are grouped in the same class, and, therefore, the approach predicts *Normal/Pathological* and *COVID-19* categories.

#### 3) Normal Vs Pathological Vs COVID-19 Approach

Additionally, we adapted a network architecture to specifically determine the degree of separability between the 3 categories of chest X-ray images considered in this work. To this end, we trained a model with a set of chest X-ray images of the 3 different categories: patients infected with COVID-19, patients with other pathologies with similar characteristics to COVID-19 and normal patients.

### C. Network Architecture

Over the recent years, deep learning methods have demonstrated to be an excellent technique in the area of artificial intelligence given its potential and ability to recognize complex patterns from raw input data, learning proper hierarchical representations of the underlying information at different levels. The great potential of these techniques is increasingly used in many relevant fields of medical imaging [48], [49], including computer-aided detection/diagnosis systems and medical image analysis for accurate early detection, diagnosis, treatment and monitoring of many relevant diseases [50], [51].

In this work, we exploit the potential of a densely convolutional network architecture inspired by the DenseNet proposal of Huang *et al.* [52], which was adapted to our issue due to its flexibility and simplicity and its preceding promising results in other classification tasks applied to the diagnosis of pulmonary diseases [53]–[55]. For this research, the original structure of the DenseNet-161 architecture has been adapted, as depicted in Table 1. Specifically, in this architecture, each layer is connected to every other layer in a feed-forward fashion within each dense block to ensure maximum information flow between layers. In order to preserve the feed-forward nature, each layer processes feature maps from all the preceding layers as separate inputs and transfers its own feature maps to all subsequent layers. The classification layer has been adapted to support the output defined for each deep learning approach considered by this proposal, that is, the categorization of chest X-ray images into 2 clinical classes, considering normal or pathological categories in the first approach and Non-COVID-19 or COVID-19 categories, in the second approach.

**TABLE 1:** An Illustration of the DenseNet-161 Structure That Was Adapted for the Experiments of This Work

Layers	Convolution	Pooling	Dense block	Transition layer		Dense block	Transition layer		Dense block	Transition layer		Dense block	Classification layer	
Output Size	$112\times 112$	$56\times 56$	$56\times 56$	$56\times 56$	$28\times 28$	$28\times 28$	$28\times 28$	$14\times 14$	$14\times 14$	$14\times 14$	$7\times 7$	$7\times 7$	$1\times 1$	–
Structure	$7\times 7$ Convolution Stride 2	$3\times 3$ Maxpool Stride 2	$\begin{aligned} \begin{bmatrix} 1 \times 1 & \text {conv} \\ 3 \times 3 & \text {conv} \end{bmatrix}\times 6 \end{aligned}$	$1\times 1$ Convolution	$2\times 2$ Average pool Stride 2	$\begin{aligned} \begin{bmatrix} 1 \times 1 & \text {conv} \\ 3 \times 3 & \text {conv} \end{bmatrix}\times 12 \end{aligned}$	$1\times 1$ Convolution	$2\times 2$ Average pool Stride 2	$\begin{aligned} \begin{bmatrix} 1 \times 1 & \text {conv} \\ 3 \times 3 & \text {conv} \end{bmatrix}\times 36 \end{aligned}$	$1\times 1$ Convolution	$2\times 2$ Average pool Stride 2	$\begin{aligned} \begin{bmatrix} 1 \times 1 & \text {conv} \\ 3 \times 3 & \text {conv} \end{bmatrix}\times 24 \end{aligned}$	$7\times 7$ Global average pool	Fully-connected softmax 2/3 classes

### D. Training Details

The dataset was randomly divided into three mutually exclusive sets, trying to balance the number of chest X-ray images associated with each category throughout the sets. In particular, this partition includes 60% of the cases for training, 20% for validation, and the remaining 20% for testing.

For the training procedure, the weights from a model pretrained on the ImageNet dataset have been used for the dense network architecture initialization [56]. Thus, the pretrained model allows a more efficient learning process and fast convergence since the weights are initially stabilized. Additionally, it significantly reduces the amount of labeled data required for an adequate model training, as is our case.

Each presented deep learning approach is trained end-to-end with a cross-entropy loss function [57] over the output class and the ground truth for the target chest X-ray image. The optimization during the training stage is performed by means of Stochastic Gradient Descent (SGD) with a constant learning rate of 0.01, a mini-batch size of 4, and a first-order momentum of 0.9. These values that were obtained under different exhaustive experimentation are directly indicated for simplicity. This optimizer, despite its simplicity, is highly efficient for the discriminative learning of classifiers under convex loss functions [58], defined in Equation 1:

\begin{equation*} L = {-} Y \cdot \text {log} (\hat {Y}) \tag{1}\end{equation*}

where $Y$ represents the ground truth values and $\hat {Y}$ represents the estimated values for each identified category. A complete training epoch includes a forward pass of all the target frames composing the training set. Each training process was performed throughout 200 epochs given that a higher number of epochs showed no significant improvements in both cross-entropy loss and classification accuracy performances.

In order to assess the generalization capability of the presented deep learning approaches to extrapolate to unseen chest X-ray images, 5 independent repetitions with a different random selection of the sample divisions were performed, being calculated the mean cross-entropy loss and the mean accuracy to evaluate their global performance.

#### 1) Data Augmentation

Despite the pretraining, given the dimensionality of the dataset, data augmentation was performed in the experiments, increasing the number of the training samples in order to avoid overfitting and improve the generalization of the network, obtaining more robust and stable trained models [59], [60].

Thus, given the limited amount of COVID-19 subjects, different configurations of affine transformations are applied to the input chest X-ray images to increase the variability of the training data and improve the performance of the convolutional neural networks. In particular, considering the possible resolutions provided by the common variety of acquisition devices as well as the symmetrical nature of the human body, several combinations of scaling with horizontal flipping operations were included throughout the training procedure.

### E. Evaluation

The performance of the presented approaches was evaluated by comparing the predictions provided by the networks with the ground truth labels annotated in the dataset. Then, using as reference the True Positives (TP), True Negatives (TN), False Positives (FP) and False Negatives (FN) extracted from this comparison, several performance metrics, commonly used in the literature to evaluate the suitability of computational methods for medical imaging, were considered. Thus, Precision, Recall, F1-score, and Accuracy were computed for each approach as follows:

\begin{align*} \text {Precision}=&\frac {\text {TP}}{\text {TP} + \text {FP}} \tag{2}\\ \text {Recall}=&\frac {\text {TP}}{\text {TP} + \text {FN}} \tag{3}\\ \text {F1-score}=&{2} \cdot \frac {\text {Precision} \cdot \text {Recall}}{\text {Precision} + \text {Recall}} \tag{4}\\ \text {Accuracy}=&\frac {\text {TP} + \text {TN}}{\text {TP} + \text {TN}} + \text {FP} + \text {FN} \tag{5}\end{align*}

## III. Experimental Results

In this section, we present the experimental results of the proposed approaches for the classification of COVID-19 in chest X-ray images. In order to explore the full potential of the available dataset, 3 experiments that complement each other were considered. The following sections detail the results for each of the experiments.

### A. 1^st^ Experiment: Analyzing the Normal Vs Pathological/COVID-19 Approach

**FIGURE 3.**
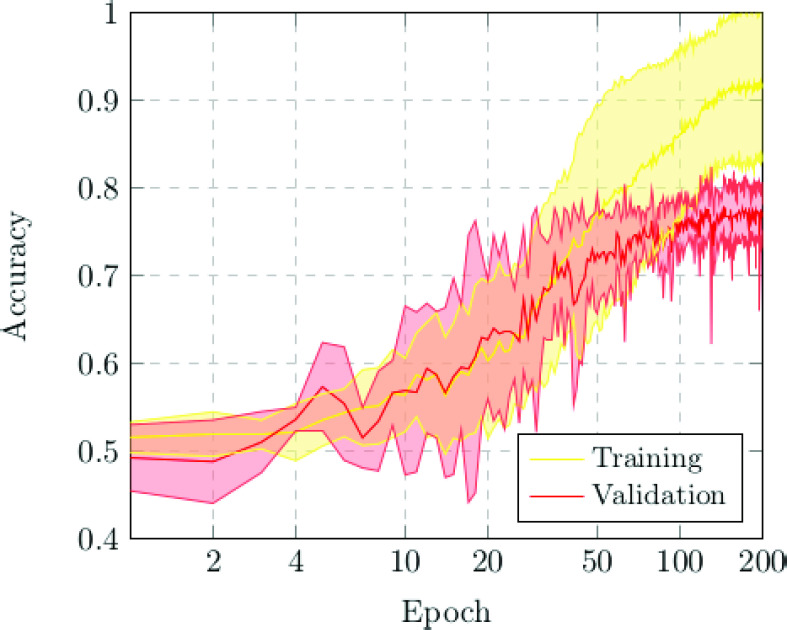
Mean ± standard deviation training and validation accuracy after the 5 independent repetitions of the first experiment. A logarithmic scale has been set to correctly display the values for a better understanding of the results.

**FIGURE 4.**
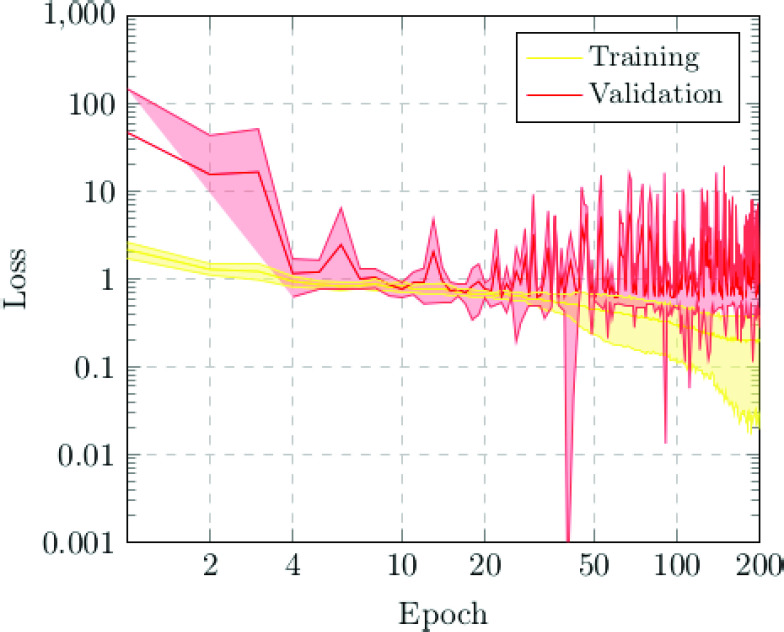
Mean ± standard deviation training and validation loss after the 5 independent repetitions of the first experiment. A logarithmic scale has been set to correctly display the values for a better understanding of the results.

The first scenario was designed in order to evaluate the performance of the proposed approach to distinguish between chest X-ray images of subjects with COVID-19 from other similar pulmonary or pleural pathological conditions as well as other non-related normal subjects. Accordingly, we designed an experiment with a total of 1,616 chest X-ray images, being 240 from COVID-19, 648 from patients with other similar lung diseases, and 728 from normal subjects. Therefore, the binary classes Normal and Pathological/COVID-19 are balanced, maintaining this proportion in the random divisions in training, validation and testing sets. Figs. 3 and 4 show the performance that was achieved using the DenseNet architecture after 5 independent repetitions in the training and validation stages in terms of mean ± standard deviation training accuracy and mean ± standard deviation training loss, respectively.

In particular, as we can observe in Fig. 3, the best mean accuracy obtained is 0.9219 ± 0.0810 for the training stage in epoch 194 and the best average accuracy for the validation stage of 0.7783 ± 0.0260 in epoch 144. Additionally, the proposed approach achieved its stability in the cross-entropy function loss both for training and for validation after epoch 150, as we can see in Fig. 4.

**FIGURE 5.**
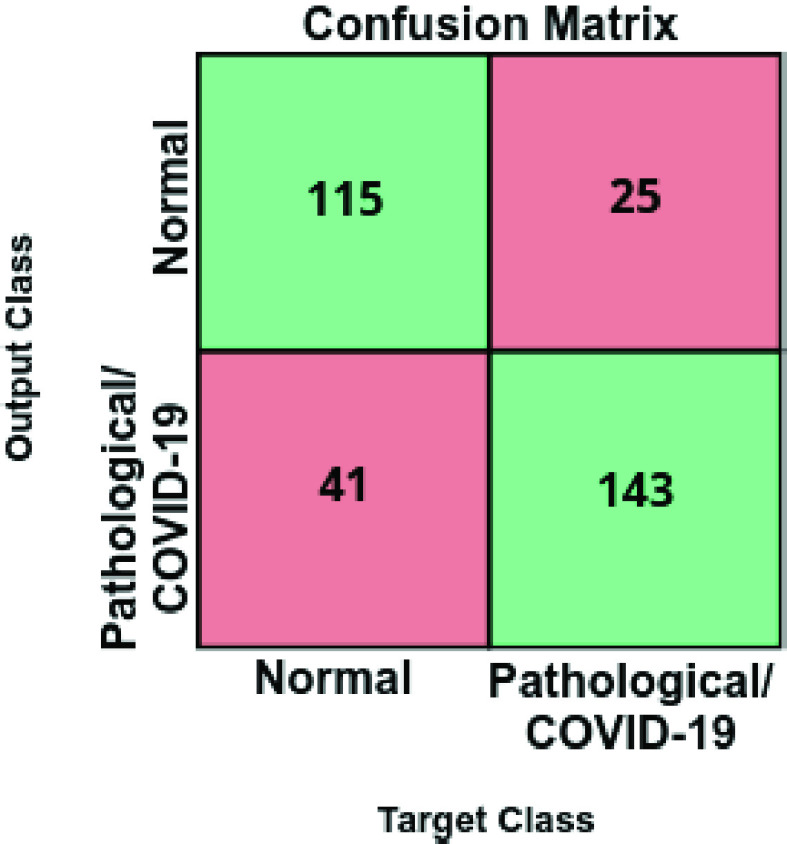
Confusion matrix for the classification of chest X-ray images between Normal vs Pathological/COVID-19 cases at the test stage.

Table 2 presents a comparative analysis of the performance at the test stage using precision, recall, and F1-score measures whereas Fig. 5 shows the corresponding confusion matrix. As we can see, the proposed strategy achieves a global accuracy of 79.62% using all the chest X-ray images of the analyzed test dataset.

**TABLE 2:** Precision, Recall, and F1-Score Results Obtained at the Test Stage for the Classification of Chest X-Ray Images Between Normal vs Pathological/COVID-19 Cases

Cases	Precision	Recall	F1-score
Normal	0.74	0.82	0.78
Pathological/COVID-19	0.85	0.78	0.81

We would like to remark that the pathological combined with the COVID-19 cases present a variety of diseases that presents a significant variability of conditions in the lungs. Also, normal subjects are those from healthy patients but also from other pathological scenarios with characteristics different from COVID-19. Given that, we have to consider the significant variability of characteristics that are represented in both considered cases of this approach. As said, given those circumstances as well as the low quality of the chest X-ray images captured by portable devices, we consider that this proposal is able to adequately distinguish pulmonary pathological cases from normal patients.

### B. 2^nd^ Experiment: Analyzing the Normal/Pathological Vs COVID-19 Approach

In this second experiment, we designed a complementary scenario to evaluate the ability of the second analyzed approach to distinguish between cases of patients with COVID-19 from the rest of similar respiratory diseases or even normal patients.

With this in mind, the proposed approach was validated using a dataset composed of 720 chest X-ray images, being 240 from normal patients whereas other 240 from pulmonary diseases and 240 from COVID-19 patients, respectively. This way, we respect a proportion of $\frac {2}{3}$ and $\frac {1}{3}$ for the Normal/ Pathological and COVID-19 classes, trying to keep a minimum of balance between both negative and positive classes of this analyzed approach. These proportions were maintained for the random divisions in the training, validation and testing sets. In this case, those subjects with pulmonary diseases and normal patients were randomly selected from the total available in the used image dataset.

**FIGURE 6.**
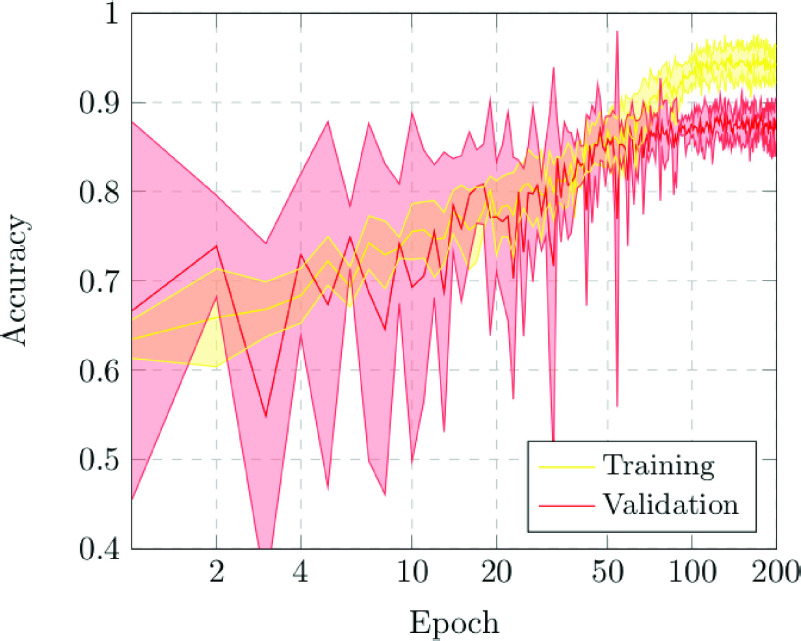
Mean ± standard deviation training and validation accuracy after the 5 independent repetitions of the second experiment. A logarithmic scale has been set to correctly display the values for a better understanding of the results.

**FIGURE 7.**
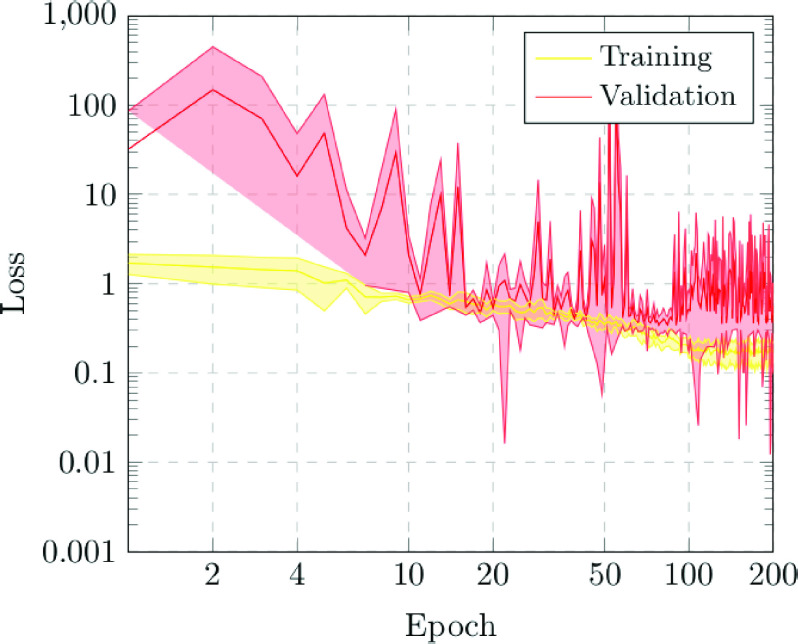
Mean ± standard deviation training and validation loss after the 5 independent repetitions of the second experiment. A logarithmic scale has been set to correctly display the values for a better understanding of the results.

Figs. 6 and 7 illustrate the performance that was obtained from the proposed deep neural architecture after 5 independent repetitions in both the training and validation stages. In particular, the proposed approach achieves a best average accuracy of 0.9550 ± 0.0204 for training in epoch 166 and the best average accuracy for validation of 0.8902 ± 0.0158 in epoch 131, as we can see in Fig. 6.

**FIGURE 8.**
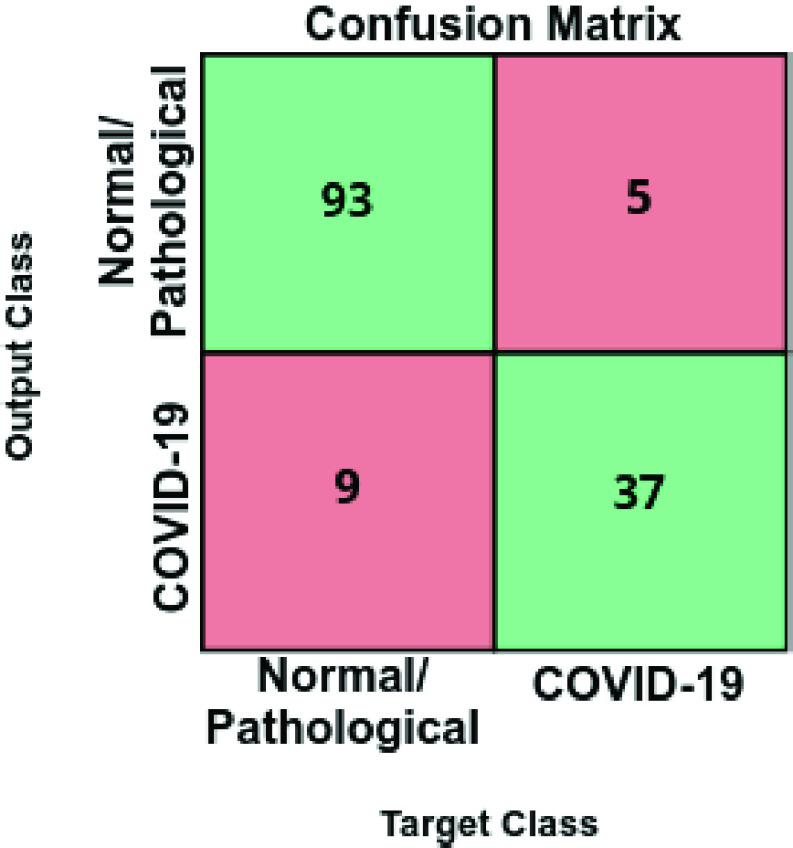
Confusion matrix for the classification of chest X-ray images between Normal/Pathological vs COVID-19 cases at the test stage.

In addition, the proposed approach achieved its stability in the loss cross-entropy function both for training and for validation after epoch 100, as we can see in Fig. 7.

Table 3 summarizes the performance measurements that were obtained by the proposed approach using the test dataset, in terms of precision, recall, and F1-score for each category, whereas Fig. 6 shows the corresponding confusion matrix. As we can see, our approach shows a good performance for both class Normal/Pathological and class COVID-19, providing a global accuracy of 90.27%. In general, the results are in line with the training performance being the proposed approach able to classify chest X-ray images between cases of patients with COVID-19 from other similar pulmonary pathologies or normal subjects.

**TABLE 3:** Precision, Recall and F1-Score Results Obtained at the Test Stage for the Classification of Chest X-Ray Images Between Normal/Pathological vs COVID-19 Cases

Cases	Precision	Recall	F1-score
Normal/Pathological	0.91	0.95	0.93
COVID-19	0.88	0.80	0.84

In this case, we have to consider that the separability is more feasible. In particular, the positive class only contains COVID-19 patients, with more restricted characteristics, being the network capable of distinguishing them from the rest of the subjects, outperforming the results of the first approach.

### C. 3^rd^ Experiment: Analyzing the Normal Vs Pathological Vs COVID-19 Approach

**FIGURE 9.**
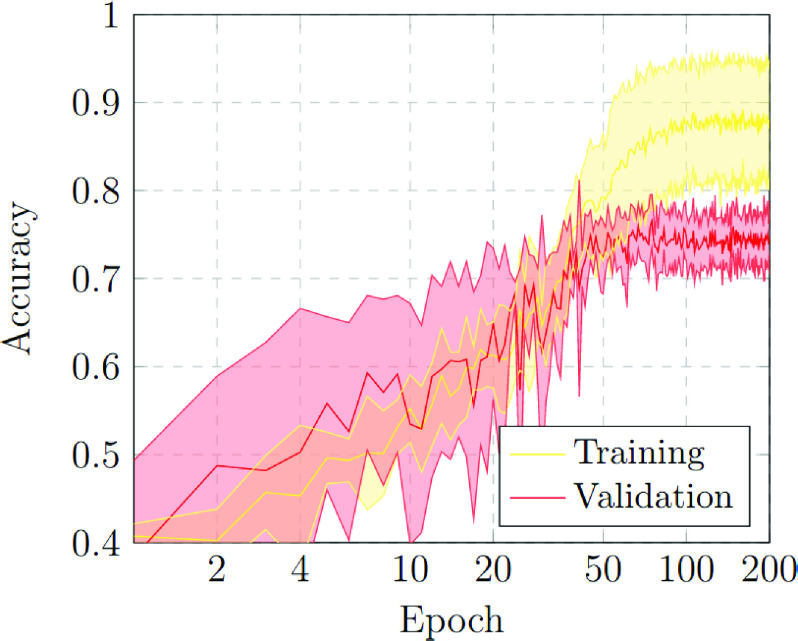
Mean ± standard deviation training and validation accuracy after the 5 independent repetitions of the third experiment. A logarithmic scale has been set to correctly display the values for a better understanding of the results.

**FIGURE 10.**
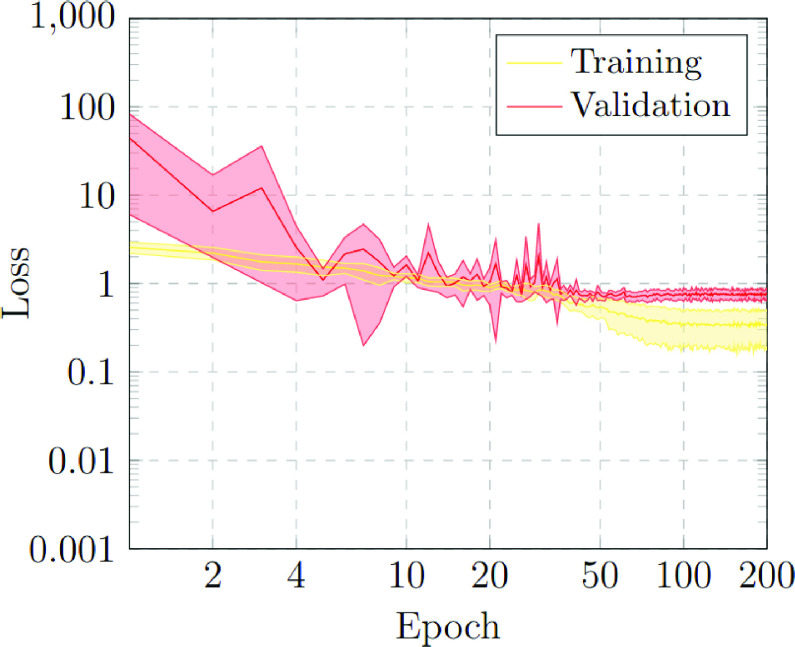
Mean ± standard deviation training and validation loss after the 5 independent repetitions of the third experiment. A logarithmic scale has been set to correctly display the values for a better understanding of the results.

The third scenario was designed in order to evaluate the performance of the proposed approach to distinguish between the 3 categories of chest X-ray images considered in this work. In particular, we designed a complete experiment using a total of 720 chest X-ray images, being 240 from COVID-19, 240 from patients with other similar lung or pleural diseases, and 240 from normal subjects. The balance in the number of chest X-ray images associated with each category was kept for the random divisions in the training, validation and testing sets. Figs. 9 and 10 show the performance that was achieved using the network architecture after 5 independent repetitions in the training and validation stages. As we can see, the best mean accuracy that was produced is 0.8907 ± 0.0546 for the training stage in epoch 160 and the best average accuracy for validation of 0.7652 ± 0.0304 in epoch 75.

**FIGURE 11.**
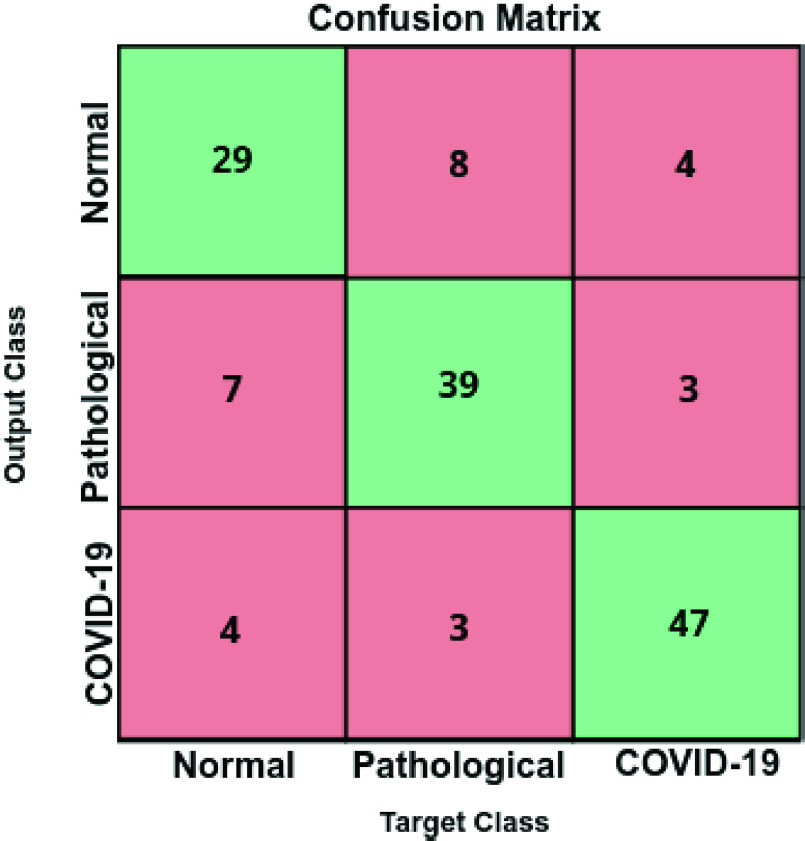
Confusion matrix for the classification of chest X-ray images between Normal vs Pathological vs COVID-19 cases at the test stage.

In Table 4, we can see the precision, recall, and F1-score results obtained at the test stage, providing a global accuracy of 79.86%. Complementary, Fig. 11 presents the confusion matrix for the classification of chest X-ray images between normal vs pathological vs COVID-19 cases at the test stage. As we can see, all the results obtained show the robustness of the proposed system in the classification of the 3 categories of chest X-ray images considered in this work.

**TABLE 4:** Precision, Recall and F1-Score Results Obtained at the Test Stage for the Classification of Chest X-Ray Images Between Normal vs Pathological vs COVID-19 Cases

Cases	Precision	Recall	F1-score
Normal	0.72	0.71	0.72
Pathological	0.78	0.80	0.79
COVID-19	0.87	0.87	0.87

## IV. Discussion

Chest X-ray has become the first-line imaging modality used for the identification of patients with COVID-19 infections at an early stage when clinical symptoms may be nonspecific or sparse. In this context, portable radiography devices are widely used by clinical specialists to evaluate suspected or confirmed cases of COVID-19 disease directly on-site, without the necessity of transferring patients potentially infected by COVID-19 elsewhere. Therefore, this clinical procedure helps to save critical time and resources for the therapeutic process as well as reduce the risk of cross-infection.

**FIGURE 12.**
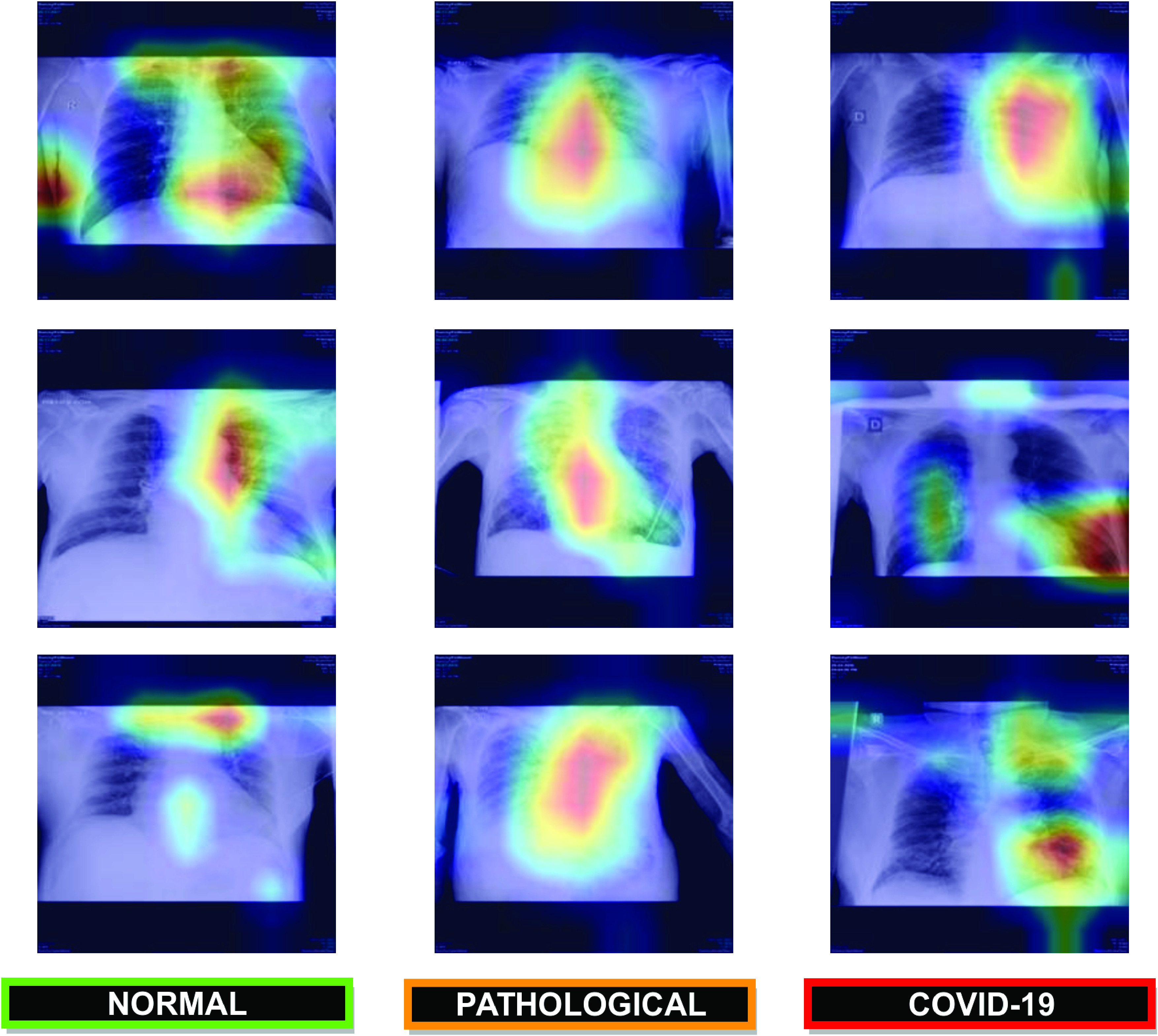
Representative examples of the generated gradient class activation maps based on predictions that illustrate parts of the chest X-ray image that are strongly activated. $1^{st}$ row, using the Normal vs Pathological/COVID-19 approach. $2^{nd}$ row, using the Normal/Pathological vs COVID-19 approach. $3^{rd}$ row, using the Normal vs Pathological vs COVID-19 approach.

In order to provide a visual interpretation of the proposed approaches, we used the Gradient Class Activation Map (Grad-CAM) [61] algorithm to generate the class activation maps of the trained models, showing us what the neural network sees and what it values when making its prediction. As a result, the trained models were shown to be strongly correlated with the clinical findings, as validated by the clinical experts. In Fig. 12, we can see some representative examples of the generated maps based on predictions that illustrate parts of the chest X-ray image that are strongly activated, as well as a considerable variability of the possible scenarios represented in this research work such as the poor contrast of the radiographs or the great similarity between COVID-19 and others pulmonary diseases that are present in our dataset.

To the best of our knowledge, these are the first fully automatic approaches that were designed specifically for the classification of COVID-19 using chest X-ray images acquired only by portable equipment. Therefore, we cannot make a direct comparison with other state-of-the-art methods.

In all scenarios, we have to consider the complexity of analyzing chest X-ray images capture by portable devices, which present a lower quality in comparison with other typically used images in many pulmonary computational proposals using X-ray images. This poor quality is combined with the complex tested scenarios of the dataset, considering images from pulmonary conditions that may be confused with the characteristics of COVID-19 as well as normal subjects that include healthy subjects but also may include other pathological conditions that are not related with the characteristics of COVID-19. As said, the designed dataset presents cases that are very frequent in real clinical practice scenarios.

Considering these complex scenarios of differentiation and the poor quality of the analyzed images, the capabilities of the adapted convolutional neural network offered an adequate performance, distinguishing specifically the COVID-19 cases from the rest, but also differentiating both COVID-19 and pulmonary pathological cases from other normal cases, a complex scenario with large variability in both negative and positive classes.

## V. Limitations of the Study

The use of portable equipment implies a greater challenge for the automatic diagnostic of the COVID-19, given that the acquired images present lower quality, being in the supine position, providing a single anterior-posterior view, unlike radiological analysis carried out in conventional rooms that supplies 2 projections, anterior-posterior and lateral. Despite the poor quality conditions of chest X-ray images acquired by portable equipment, the obtained results show that the proposed approaches allow a robust and reliable analysis to support the clinical decision-making process in a real public health emergency scenario. Despite this, the method presents some intrinsic limitations due to the complex characteristics that may be present in the X-ray images. Some cases of misclassification are due to poor contrast of X-ray images or the presence of medical instruments such as endotracheal tubes, orthopedic prosthetic devices, cardiac pacemakers, among others. With an eye to the future, it would be necessary to obtain more X-ray images including these specific cases in order to provide a more comprehensive validation of the proposed methodology.

## VI. Conclusion

Coronavirus disease (COVID-19), caused by the newly discovered severe acute respiratory syndrome coronavirus 2 (SARS-CoV-2) is posing high pressure on healthcare services worldwide given its drastic spreading. Therefore, the screening, early detection, and monitoring of the suspected individuals are crucial to controlling the virus transmission. Given the impact of COVID-19 in the pulmonary tissues, chest radiography plays a main role in the examination of the manifestations and patterns of lung abnormality associated with the disease in order to support the diagnosis and determine its severity. Furthermore, in the context of this global pandemic, the use of portable equipment for the acquisition of chest X-ray images represents the first and main radiological way of analysis due to the more effective separation of the infected patients, allowing to minimize the infection control issues and, therefore, the risk of cross-contamination.

Thus, this work proposes a novel fully automatic methodology for the analysis of chest X-ray images acquired by means of portable devices. Given the similarities between COVID-19 and other pulmonary diseases that also affect the lungs, the proposed methodology is composed of three complementary deep learning approaches in order to better analyze and differentiate different patients infected with COVID-19, patients with other pathologies with similar characteristics to COVID-19 and normal patients. Thus, the first approach allows to classify between normal and pathological patients, being pathological both patients with COVID-19 and patients with other similar respiratory conditions. The second approach is aimed at differentiating between patients with COVID-19 and patients that may be normal or present respiratory conditions other than COVID-19. The third approach allows the classification between the 3 defined clinical categories, simultaneously. This way, the joint response of all the approaches allows to classify and better analyze the considered COVID-19, pathological and normal cases.

The proposed methodology and the tested approaches were validated using a dataset provided by the Radiology Service of the Complexo Hospitalario Universitario A Coruña (CHUAC). To the best of our knowledge, it is the first proposal specifically tailored for the classification of chest X-ray images acquired exclusively by portable equipment. Despite the poor quality conditions of these chest X-ray images, the obtained results show that the proposed methodology and the tested approaches allow a robust and reliable analysis to support the clinical decision-making process in this context of a public health emergency.

As future work, we plan to test the potential of the proposed approaches using datasets acquired by portable equipment, when publicly available, to provide a more complete validation with a screening scenario similar to that studied in this work.

## References

[1] K. McIntosh. (2020). Coronavirus Disease 2019 (COVID-19): Epidemiology, Virology, Clinical Features, Diagnosis, and Prevention. Accessed: Oct. 11, 2020. [Online]. Available: https://www.uptodate.com/contents/coronavirus-disease-2019-covid-19-epidemiology-virology-clinical-features-diagnosis-and-prevention

[2] C. Wang, P. W. Horby, F. G. Hayden, and G. F. Gao, “A novel coronavirus outbreak of global health concern,” Lancet, vol. 395, no. 10223, pp. 470–473, Feb. 2020. 31986257 10.1016/S0140-6736(20)30185-9 PMC7135038

[3] World Health Organisation. (2020). WHO Director-General’s Opening Remarks at the Media Briefing on COVID-19–11 March 2020. Accessed: Oct. 11, 2020. [Online]. Available: https://www.who.int/dg/speeches/detail/who-director-general-s-opening-remarks-at-the-media-briefing-on-covid-19-11-march-2020

[4] World Health Organisation. (2020). Coronavirus Disease (COVID-2019) Situation Reports—Situation Report—114. Accessed: Oct. 11, 2020. [Online]. Available: https://www.who.int/emergencies/diseases/novel-coronavirus-2019/situation-reports

[5] A. Biswas, U. Bhattacharjee, A. K. Chakrabarti, D. N. Tewari, H. Banu, and S. Dutta, “Emergence of novel coronavirus and COVID-19: Whether to stay or die out?” Crit. Rev. Microbiol., vol. 46, no. 2, pp. 182–193, Mar. 2020. 32282268 10.1080/1040841X.2020.1739001 PMC7157960

[6] V. Grech, “Unknown unknowns—COVID-19 and potential global mortality,” Early Hum. Develop., vol. 144, 5 2020, Art. no. 105026. 10.1016/j.earlhumdev.2020.105026 PMC7270771 32247898

[7] C.-C. Lai, T.-P. Shih, W.-C. Ko, H.-J. Tang, and P.-R. Hsueh, “Severe acute respiratory syndrome coronavirus 2 (SARS-CoV-2) and corona virus disease-2019 (COVID-19): The epidemic and the challenges,” Int. J. Antimicrobial Agents, vol. 55, Mar. 2020, Art. no. 105924. 10.1016/j.ijantimicag.2020.105924 PMC7127800 32081636

[8] M. O. Wielpütz, C. P. Heußel, F. J. F. Herth, and H.-U. Kauczor, “Radiological diagnosis in lung disease,” Deutsches Aerzteblatt Online, vol. 111, no. 11, pp. 181–187, Mar. 2014. 10.3238/arztebl.2014.0181 PMC3977441 24698073

[9] W. C. Black and H. G. Welch, “Advances in diagnostic imaging and overestimations of disease prevalence and the benefits of therapy,” New England J. Med., vol. 328, no. 17, pp. 1237–1243, Apr. 1993, doi: 10.1056/NEJM199304293281706. 8464435

[10] S. Candemir and S. Antani, “A review on lung boundary detection in chest X-rays,” Int. J. Comput. Assist. Radiol. Surg., vol. 14, no. 4, pp. 563–576, Apr. 2019. 30730032 10.1007/s11548-019-01917-1 PMC6420899

[11] W. Ausawalaithong, A. Thirach, S. Marukatat, and T. Wilaiprasitporn, “Automatic lung cancer prediction from chest X-ray images using the deep learning approach,” in Proc. 11th Biomed. Eng. Int. Conf. (BMEiCON), Nov. 2018, pp. 1–5.

[12] W.-J. Guan, “Clinical characteristics of coronavirus disease 2019 in China,” New England J. Med., vol. 382, no. 18, pp. 1708–1720, Apr. 2020, doi: 10.1056/NEJMoa2002032. 32109013 PMC7092819

[13] H. Y. F. Wong, H. Y. S. Lam, A. H.-T. Fong, S. T. Leung, T. W.-Y. Chin, C. S. Y. Lo, M. M.-S. Lui, J. C. Y. Lee, K. W.-H. Chiu, T. W.-H. Chung, E. Y. P. Lee, E. Y. F. Wan, I. F. N. Hung, T. P. W. Lam, M. D. Kuo, and M.-Y. Ng, “Frequency and distribution of chest radiographic findings in COVID-19 positive patients,” Radiology, vol. 296, Mar. 2020, Art. no. 201160. 10.1148/radiol.2020201160 PMC7233401 32216717

[14] C. Long, H. Xu, Q. Shen, X. Zhang, B. Fan, C. Wang, B. Zeng, Z. Li, X. Li, and H. Li, “Diagnosis of the coronavirus disease (COVID-19): rRT-PCR or CT?” Eur. J. Radiol., vol. 126, 5 2020, Art. no. 108961. 10.1016/j.ejrad.2020.108961 PMC7102545 32229322

[15] M. Kim, J. Yun, Y. Cho, K. Shin, R. Jang, H.-J. Bae, and N. Kim, “Deep learning in medical imaging,” Neurospine, vol. 16, no. 4, pp. 657–668, 2019. 31905454 10.14245/ns.1938396.198 PMC6945006

[16] M. M. A. Monshi, J. Poon, and V. Chung, “Deep learning in generating radiology reports: A survey,” Artif. Intell. Med., vol. 106, Jun. 2020, Art. no. 101878. 10.1016/j.artmed.2020.101878 PMC7227610 32425358

[17] Y.-X. Tang, Y.-B. Tang, Y. Peng, K. Yan, M. Bagheri, B. A. Redd, C. J. Brandon, Z. Lu, M. Han, J. Xiao, and R. M. Summers, “Automated abnormality classification of chest radiographs using deep convolutional neural networks,” npj Digit. Med., vol. 3, no. 1, pp. 1–8, Dec. 2020. 32435698 10.1038/s41746-020-0273-z PMC7224391

[18] M. Anthimopoulos, S. Christodoulidis, L. Ebner, A. Christe, and S. Mougiakakou, “Lung pattern classification for interstitial lung diseases using a deep convolutional neural network,” IEEE Trans. Med. Imag., vol. 35, no. 5, pp. 1207–1216, 5 2016. 10.1109/TMI.2016.2535865 26955021

[19] M. I. Campo, J. Pascau, and R. S. J. Estépar, “Emphysema quantification on simulated X-rays through deep learning techniques,” in Proc. IEEE 15th Int. Symp. Biomed. Imag. (ISBI), Apr. 2018, pp. 273–276. 10.1109/ISBI.2018.8363572 PMC6239425 30450153

[20] A. K. Jaiswal, P. Tiwari, S. Kumar, D. Gupta, A. Khanna, and J. J. P. C. Rodrigues, “Identifying pneumonia in chest X-rays: A deep learning approach,” Measurement, vol. 145, pp. 511–518, Oct. 2019.

[21] F. Pasa, V. Golkov, F. Pfeiffer, D. Cremers, and D. Pfeiffer, “Efficient deep network architectures for fast chest X-ray tuberculosis screening and visualization,” Sci. Rep., vol. 9, no. 1, Dec. 2019, Art. no. 6268. 10.1038/s41598-019-42557-4 PMC6472370 31000728

[22] V. Chouhan, S. K. Singh, A. Khamparia, D. Gupta, P. Tiwari, C. Moreira, R. Damaševičius, and V. H. C. de Albuquerque, “A novel transfer learning based approach for pneumonia detection in chest X-ray images,” Appl. Sci., vol. 10, no. 2, p. 559, Jan. 2020.

[23] I. D. Apostolopoulos, S. I. Aznaouridis, and M. A. Tzani, “Extracting possibly representative COVID-19 biomarkers from X-ray images with deep learning approach and image data related to pulmonary diseases,” J. Med. Biol. Eng., vol. 40, pp. 1–8, 5 2020. 10.1007/s40846-020-00529-4 PMC7221329 32412551

[24] L. Wang and A. Wong, “COVID-Net: A tailored deep convolutional neural network design for detection of COVID-19 cases from chest X-ray images,” 2020, arXiv:2003.09871. [Online]. Available: http://arxiv.org/abs/2003.09871 10.1038/s41598-020-76550-z PMC7658227 33177550

[25] K. Hammoudi, H. Benhabiles, M. Melkemi, F. Dornaika, I. Arganda-Carreras, D. Collard, and A. Scherpereel, “Deep learning on chest X-ray images to detect and evaluate pneumonia cases at the era of COVID-19,” 2020, arXiv:2004.03399. [Online]. Available: http://arxiv.org/abs/2004.03399 10.1007/s10916-021-01745-4 PMC8185498 34101042

[26] E. E.-D. Hemdan, M. A. Shouman, and M. E. Karar, “COVIDX-Net: A framework of deep learning classifiers to diagnose COVID-19 in X-ray images,” 2020, arXiv:2003.11055. [Online]. Available: http://arxiv.org/abs/2003.11055

[27] J. de Moura, J. Novo, and M. Ortega, “Fully automatic deep convolutional approaches for the analysis of Covid-19 using chest X-ray images,” medRxiv, pp. 1–13, 5 2020. [Online]. Available: https://www.medrxiv.org/content/early/2020/05/06/2020.05.01.20087254, doi: 10.1101/2020.05.01.20087254. PMC8645263 34899109

[28] J. Zhang, Y. Xie, G. Pang, Z. Liao, J. Verjans, W. Li, Z. Sun, J. He, Y. Li, C. Shen, and Y. Xia, “Viral pneumonia screening on chest X-ray images using confidence-aware anomaly detection,” 2020, arXiv:2003.12338. [Online]. Available: http://arxiv.org/abs/2003.12338 10.1109/TMI.2020.3040950 PMC8544953 33245693

[29] T. Ozturk, M. Talo, E. A. Yildirim, U. B. Baloglu, O. Yildirim, and U. R. Acharya, “Automated detection of COVID-19 cases using deep neural networks with X-ray images,” Comput. Biol. Med., vol. 121, Jun. 2020, Art. no. 103792. 10.1016/j.compbiomed.2020.103792 PMC7187882 32568675

[30] A. Shelke, M. Inamdar, V. Shah, A. Tiwari, A. Hussain, T. Chafekar, and N. Mehendale, “Chest X-ray classification using Deep learning for automated COVID-19 screening,” medRxiv, pp. 1–19, Jun. 2020. [Online]. Available: https://www.medrxiv.org/content/early/2020/06/23/2020.06.21.20136598, doi: 10.1101/2020.06.21.20136598. PMC8152712 34075355

[31] S. H. Yoo, H. Geng, T. L. Chiu, S. K. Yu, D. C. Cho, J. Heo, M. S. Choi, I. H. Choi, C. C. Van, N. V. Nhung, B. J. Min, and H. Lee, “Deep learning-based decision-tree classifier for COVID-19 diagnosis from chest X-ray imaging,” Frontiers Med., vol. 7, p. 427, Jul. 2020. 10.3389/fmed.2020.00427 PMC7371960 32760732

[32] M. D. Li, N. T. Arun, M. Gidwani, K. Chang, F. Deng, B. P. Little, D. P. Mendoza, M. Lang, S. I. Lee, A. O’Shea, A. Parakh, P. Singh, and J. Kalpathy-Cramer, “Automated assessment and tracking of COVID-19 pulmonary disease severity on chest radiographs using convolutional siamese neural networks,” Radiol., Artif. Intell., vol. 2, no. 4, Jul. 2020, Art. no. e200079. 10.1148/ryai.2020200079 PMC7392327 33928256

[33] A. I. Khan, J. Shah, and M. Bhat, “CoroNet: A deep neural network for detection and diagnosis of COVID-19 from chest X-ray images,” Comput. Methods Programs Biomed., vol. 196, Nov. 2020, Art. no. 105581. 10.1016/j.cmpb.2020.105581 PMC7274128 32534344

[34] A. Waheed, M. Goyal, D. Gupta, A. Khanna, F. Al-Turjman, and P. R. Pinheiro, “CovidGAN: Data augmentation using auxiliary classifier GAN for improved COVID-19 detection,” IEEE Access, vol. 8, pp. 91916–91923, 2020. 34192100 10.1109/ACCESS.2020.2994762 PMC8043420

[35] A. Narin, C. Kaya, and Z. Pamuk, “Automatic detection of coronavirus disease (COVID-19) using X-ray images and deep convolutional neural networks,” 2020, arXiv:2003.10849. [Online]. Available: http://arxiv.org/abs/2003.10849 10.1007/s10044-021-00984-y PMC8106971 33994847

[36] D. Dansana, R. Kumar, A. Bhattacharjee, D. J. Hemanth, D. Gupta, A. Khanna, and O. Castillo, “Early diagnosis of COVID-19-affected patients based on X-ray and computed tomography images using deep learning algorithm,” Soft Comput., pp. 1–9, Aug. 2020, doi: 10.1007/s00500-020-05275-y. PMC7453871 32904395

[37] S. Minaee, R. Kafieh, M. Sonka, S. Yazdani, and G. J. Soufi, “Deep-COVID: Predicting COVID-19 from chest X-ray images using deep transfer learning,” Med. Image Anal., vol. 65, Oct. 2020, Art. no. 101794. 10.1016/j.media.2020.101794 PMC7372265 32781377

[38] S. Basu, S. Mitra, and N. Saha, “Deep learning for screening COVID-19 using chest X-ray images,” medRxiv, pp. 1–6, 5 2020. [Online]. Available: https://www.medrxiv.org/content/early/2020/05/08/2020.05.04.20090423, doi: 10.1101/2020.05.04.20090423.

[39] J. P. Cohen, P. Morrison, and L. Dao, “COVID-19 image data collection,” 2020, arXiv:2003.11597. [Online]. Available: http://arxiv.org/abs/2003.11597

[40] Radiological Society of North America. (2020). Radiological Society of North America (RSNA), Pneumonia Detection Challenge. Accessed: Oct. 11, 2020. [Online]. Available: https://www.kaggle.com/c/rsna-pneumonia-detection-challenge

[41] A. V. Vodovatov, I. G. Kamishanskaya, A. A. Drozdov, and C. Bernhardsson, “Quality assessment of digital X-ray chest images using an anthropomorphic chest phantom,” J. Phys., Conf. Ser., vol. 808, Feb. 2017, Art. no. 012009.

[42] D. Khezerloo, N. Gharehaghaji, and T. Abbasiazar, “Image quality assessment of the digital radiography units in tabriz, iran: A phantom study,” J. Med. Signals Sensors, vol. 9, no. 2, p. 137, 2019. 10.4103/jmss.JMSS_30_18 PMC6601229 31316908

[43] C. Martin, “Optimisation in general radiography,” Biomed. Imag. Intervent J., vol. 3, no. 2, p. e18, Apr. 2007. 10.2349/biij.3.2.e18 PMC3097657 21614270

[44] A. Jacobi, M. Chung, A. Bernheim, and C. Eber, “Portable chest X-ray in coronavirus disease-19 (COVID-19): A pictorial review,” Clin. Imag., vol. 64, pp. 35–42, Aug. 2020. 10.1016/j.clinimag.2020.04.001 PMC7141645 32302927

[45] Y. Goh, W. Chua, J. K. T. Lee, B. W. L. Ang, C. R. Liang, C. A. Tan, D. A. W. Choong, H. X. Hoon, M. K. L. Ong, and S. T. Quek, “Operational strategies to prevent coronavirus disease 2019 (COVID-19) spread in radiology: Experience from a singapore radiology department after severe acute respiratory syndrome,” J. Amer. College Radiol., vol. 17, no. 6, pp. 717–723, Jun. 2020. 10.1016/j.jacr.2020.03.027 PMC7128642 32298643

[46] American College of Radiology. (2020). ACR Recommendations for the Use of Chest Radiography and Computed Tomography (CT) for Suspected COVID-19 Infection. Accessed: Oct. 11, 2020. [Online]. Available: https://www.uptodate.com/contents/coronavirus-disease-2019-covid-19-epidemiology-virology-clinical-features-diagnosis-and-prevention

[47] Ottawa (ON): Canadian Agency for Drugs and Technologies in Health. (2016). Portable Versus Fixed X-ray Equipment: A Review of the Clinical Effectiveness, Cost-Effectiveness, and Guidelines. Accessed: Oct. 11, 2020. [Online]. Available: https://www.ncbi.nlm.nih.gov/books/NBK350586/27030858

[48] J.-G. Lee, S. Jun, Y.-W. Cho, H. Lee, G. B. Kim, J. B. Seo, and N. Kim, “Deep learning in medical imaging: General overview,” Korean J. Radiol., vol. 18, no. 4, pp. 570–584, 2017. 28670152 10.3348/kjr.2017.18.4.570 PMC5447633

[49] O. Ronneberger, P. Fischer, and T. Brox, “U-Net: Convolutional networks for biomedical image segmentation,” in Proc. Int. Conf. Med. Image Comput. Comput.-Assist. Intervent. Cham, Switzerland: Springer, 2015, pp. 234–241.

[50] J. D. Moura, J. Novo, and M. Ortega, “Deep feature analysis in a transfer learning-based approach for the automatic identification of diabetic macular edema,” in Proc. Int. Joint Conf. Neural Netw. (IJCNN), Jul. 2019, pp. 1–8.

[51] P. L. Vidal, J. de Moura, J. Novo, and M. Ortega, “Cystoid fluid color map generation in optical coherence tomography images using a densely connected convolutional neural network,” in Proc. Int. Joint Conf. Neural Netw. (IJCNN), Jul. 2019, pp. 1–8.

[52] G. Huang, Z. Liu, L. Van Der Maaten, and K. Q. Weinberger, “Densely connected convolutional networks,” in Proc. IEEE Conf. Comput. Vis. Pattern Recognit. (CVPR), Jul. 2017, pp. 4700–4708.

[53] R. Dey, Z. Lu, and Y. Hong, “Diagnostic classification of lung nodules using 3D neural networks,” in Proc. IEEE 15th Int. Symp. Biomed. Imag. (ISBI), Apr. 2018, pp. 774–778.

[54] Y. Liu, P. Hao, P. Zhang, X. Xu, J. Wu, and W. Chen, “Dense convolutional binary-tree networks for lung nodule classification,” IEEE Access, vol. 6, pp. 49080–49088, 2018.

[55] W. Guo, Z. Xu, and H. Zhang, “Interstitial lung disease classification using improved DenseNet,” Multimedia Tools Appl., vol. 78, no. 21, pp. 30615–30626, Nov. 2019.

[56] J. Deng, W. Dong, R. Socher, L.-J. Li, K. Li, and L. Fei-Fei, “ImageNet: A large-scale hierarchical image database,” in Proc. IEEE Conf. Comput. Vis. Pattern Recognit., Jun. 2009, pp. 248–255.

[57] Z. Zhang and M. Sabuncu, “Generalized cross entropy loss for training deep neural networks with noisy labels,” in Proc. Adv. Neural Inf. Process. Syst., 2018, pp. 8778–8788. PMC11747755 39839708

[58] I. Sutskever, J. Martens, G. Dahl, and G. Hinton, “On the importance of initialization and momentum in deep learning,” in Proc. Int. Conf. Mach. Learn., 2013, pp. 1139–1147.

[59] D. A. van Dyk and X.-L. Meng, “The art of data augmentation,” J. Comput. Graph. Statist., vol. 10, no. 1, pp. 1–50, Mar. 2001, doi: 10.1198/10618600152418584.

[60] Z. Hussain, F. Gimenez, D. Yi, and D. Rubin, “Differential data augmentation techniques for medical imaging classification tasks,” in Proc. AMIA Annu. Symp. Bethesda, MD, USA: American Medical Informatics Association, 2017, pp. 979–984. PMC5977656 29854165

[61] R. R. Selvaraju, M. Cogswell, A. Das, R. Vedantam, D. Parikh, and D. Batra, “Grad-CAM: Visual explanations from deep networks via gradient-based localization,” in Proc. IEEE Int. Conf. Comput. Vis. (ICCV), Oct. 2017, pp. 618–626.

